# A Pathological Condition Affects Motor Modules in a Bipedal Locomotion Model

**DOI:** 10.3389/fnbot.2019.00079

**Published:** 2019-09-20

**Authors:** Daisuke Ichimura, Tadashi Yamazaki

**Affiliations:** ^1^Graduate School of Informatics and Engineering, The University of Electro-Communications, Tokyo, Japan; ^2^Heisei Ougi Hospital, Tokyo, Japan

**Keywords:** motor module, CPG, locomotion, neuromusculoskeletal model, pathological locomotion

## Abstract

Bipedal locomotion is a basic motor activity that requires simultaneous control of multiple muscles. Physiological experiments suggest that the nervous system controls bipedal locomotion efficiently by using motor modules of synergistic muscle activations. If these modules were merged, abnormal locomotion patterns would be realized as observed in patients with neural impairments such as chronic stroke. However, sub-acute patients have been reported not to show such merged motor modules. Therefore, in this study, we examined what conditions in the nervous system merges motor modules. we built a two-dimensional bipedal locomotion model that included a musculoskeletal model with 7 segments and 18 muscles, a neural system with a hierarchical central pattern generator (CPG), and various feedback inputs from reflex organs. The CPG generated synergistic muscle activations comprising 5 motor modules to produce locomotion phases. Our model succeeded to acquire stable locomotion by using the motor modules and reflexes. Next, we examined how a pathological condition altered motor modules. Specifically, we weakened neural inputs to muscles on one leg to simulate a stroke condition. Immediately after the simulated stroke, the model did not walk. Then, internal parameters were modified to recover stable locomotion. We refitted either (a) reflex parameters or (b) CPG parameters to compensate the locomotion by adapting (a) reflexes or (b) the controller. Stable locomotion was recovered in both conditions. However the motor modules were merged only in (b). These results suggest that light or sub-acute stroke patients, who can compensate stable locomotion by just adapting reflexes, would not show merge of motor modules, whereas severe or chronic patients, who needed to adapt the controller for compensation, would show the merge, as consistent with experimental findings.

## 1. Introduction

Bipedal locomotion is a basic motor activity. Several studies suggest that animal locomotion is controlled by central pattern generators (CPGs) in the spinal cord (Grillner, 1975). CPGs provide a rhythmic motor activity across multiple muscles in a coordinated manner in both space and time (Guertin, 2009). Specifically, they generate coordinated flexor-extensor muscles' activity, and adapt gait patterns to environmental changes by using sensory feedback. The motor modules hypothesis (Dominici et al., 2011; Lacquaniti et al., 2012) proposes that the motor system groups muscles into a smaller number of functional modules based on CPGs. Physiological experiments suggest that human locomotion may involve the motor modules of synergistic muscle activations. In healthy adults, 4 or 5 motor modules are activated independently while walking (Ivanenko et al., 2004, 2005). Each module corresponds to a key phase of the gait cycle (Neptune et al., 2009). The 1st module acts to support the body in the early stance. The 2nd module acts to support both the body and propulsion in the later stance. The 3rd module contributes to decelerating the leg in the early and later part of the leg swing. The 4th module absorbs leg energy in the late part of the leg swing. Patients with neural disorders showed different combinations of motor modules, including the decrease of the number of motor modules (Ting et al., 2015). Previous studies demonstrated that locomotor rehabilitation improved walking ability in stroke patients while increasing the number of motor modules (Routson et al., 2013; Ting et al., 2015; Ferrante et al., 2016). Chronic stroke patients exhibit abnormal locomotion patterns, and 2 or more motor modules are merged in a single module, creating a timing overlap (Clark et al., 2010; Routson et al., 2013). These results suggest that motor modules are demonstrated as physiological markers of the status of patients with neural disorders (Cheung et al., 2012; Ting et al., 2015). On the contrary, some studies showed that locomotion recovery was not associated with changes in the number of motor modules (Hashiguchi et al., 2016; Tan et al., 2018). Stroke patients in the sub-acute phase showed a pattern of motor modules similar to those of healthy controls (Gizzi et al., 2011). Thus, how the nervous system in chronic stroke patients alters the motor modules remains unclear.

To address this question, we employed computer simulation of bipedal locomotion model (Taga et al., 1991; Taga, 1995; Ogihara and Yamazaki, 2001; Hase and Yamazaki, 2002; Jo and Massaquoi, 2007; Aoi et al., 2010, 2012; Allen et al., 2013). Specifically, we aimed to examine how the nervous system alters the organization of motor modules by building a two-dimensional bipedal locomotion model consisting of a musculoskeletal system and hierarchical CPGs. First, we built a normal locomotion model with 5 motor modules and various feedback inputs. By setting internal parameters by a genetic algorithm (GA) appropriately, the model succeeded to walk robustly. Then, we simulated a pathological condition. Motor evoked potentials (MEPs) are widely used to investigate the physiology of corticospinal condition (Hendricks et al., 2002). A previous study has shown a correlation between weaker lower-limb MEPs and lower gait ability (Piron et al., 2005). Therefore, we weakened output signals to the muscles on one leg to simulate the weak lower-limb MEPs. By this manipulation, the model failed to walk anymore. Then, we refit parameters by running additional GAs. Specifically, we modified one of the two sets of parameters by GAs: (a) reflex parameters to simulate patients who can compensate locomotion only by adapting reflexes, and (b) CPG parameters to simulate patients who need to adapt the controller itself for compensation. In both cases, the model succeeded to walk again, and found the only in the latter case, motor modules were merged.

## 2. Materials and Methods

### 2.1. Skeletal Model

**Figure 1:**
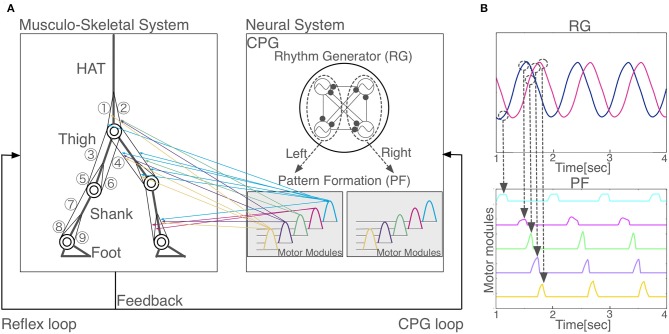
Model overview. **(A)** Schematic representation of the proposed neuromuscloskeletal model. Left panel: Musculoskeletal model. (1) gluteus maximus (GM); (2) iliopsoas (IL); (3) biceps femoris long head (BFL); (4) rectus femoris (RF); (5) biceps femoris short head (BFS); (6) vastus (VA); (7) gastrocnemius (GC); (8) soleus (SO); (9) tibia anterior (TA). HAT, head, arms, and torso. Right panel: the neural system is a central pattern generator (CPG) model, which is organized hierarchically with a rhythm generator (RG) network and a pattern formation (PF) network. Two out of the four oscillators in RG (surrounded by the left dashed circle) induce phasic and rhythmic activity patterns for five motor modules in PF that activate muscles for the left leg (colored arrows from the right to left panels), whereas the other two oscillators in RG (surrounded by the right dashed circle) induce another five motor modules in PF for the right leg (arrows are not shown). **(B)** Hierarchical relationship between RG and PF. Two neurons in RG network generate five activity patterns with different phases in PF network. When the outputs of the RG network reach certain values (dashed circles), PF neurons are activated. One PF neuron represents one motor module.

Our two-dimensional skeletal model consists of 7 rigid links that represent the head, arms, torso (HAT), thighs, shanks, and feet (Figure 1A). The length, mass and moment of inertia for each segment are taken from Jo and Massaquoi (2007) as in Table 1.

**Table 1:** Skeletal model parameters.

	**HAT**	**Thigh**	**Shank**	**Foot**
Length (m)	0.800	0.4165	0.418	0.066
Mass (kg)	44.070	6.500	3.055	0.975
Moment of inertia (kgm^2^)	5.823	0.117	0.048	9.347 ×10^−5^

Each joint is modeled as a pin joint and has a linear viscous element. The coefficients of viscosity for the hip, knee, and ankle joints are 1.09, 3.17, and 0.943 Nms/rad, respectively (Aoi et al., 2010). The knee and ankle joint are locked to prevent hyperextension or hyperflexion.

The spring and damper coefficients are 2.0 × 10^3^Nm/rad and 5.0 × 10^2^ Nms/rad for the knee joints, as well as 2.0 × 10^3^Nm/rad and 5.0 × 10^2^ Nms/rad for the ankle joint. When the heels or toes make contact with the ground, they receive the ground reaction forces produced by springs and dampers. The spring and damper coefficients are 5.0 × 10^3^N/m and 1.0 × 10^3^ Ns/m horizontally and 2.5 × 10^4^N/m and 5.0 × 10^2^ Ns/m vertically.

The equations of motion are derived by means of the Newton-Euler method (Taga et al., 1991; Taga, 1995). The general form of the equations is described as

(1) $$\begin{array}{l} \ddot{x}=P(x)F+Q(x,\dot{x},GRF(x,\dot{x}),T_{r}(F_{m})), \end{array}$$

where **x** is a (21 × 1) vector of the inertial positions of 7 links and the inertial angles of 7 links, *P* is a (21 × 12) matrix, **F** is a (8 × 1) vector of constraint forces, **Q** is a (20 × 1) vector, **GRF** is a (8 × 1) vector of ground reaction forces on the feet; **T_r_** is a (6 × 1) vector of torques, and **F_m_** (18 × 1) is a vector of the muscle tensions to be explained in the next section.

Constraint forces on the joints are eliminated by using equations of kinematic constraints given as follows:

(2) $$\begin{array}{l} C(x)\ddot{x}=D(x,\ddot{x}) \end{array}$$

where *C* is a (12 × 21) matrix and **D** is a (12 × 1) vector. By eliminating **F** from Equations (1) and (2), we obtain

(3) $$\begin{array}{l} \ddot{x}=P(x){\left[C\left(x\right)P\left(x\right)\right]}^{-1}[D\left(x,\ddot{x}\right)\\ -C\left(x\right)Q\left(x,\dot{x},GRF\left(x,\dot{x}\right),T_{r}\left(F_{m}\right)\right)]\\ +Q\left(x,\dot{x},GRF\left(x,\dot{x}\right),T_{r}\left(F_{m}\right)\right). \end{array}$$

This equation can be numerically integrated, given the muscle tensions **F_m_**.

### 2.2. Muscular Model

We used 18 principal muscles; 9 for each legs (Figure 1A): gluteus maximus (GM), iliopsoas (IL), biceps femoris long head (BFL), rectus femoris (RF), biceps femoris short head (BFS) vastus (VA), gastrocnemius (GC), soleus (SO), tibia anterior (TA). A muscle receives a signal from the corresponding α-motoneuron and generates muscle tension depending on the force-length and force-velocity relationship. We used the mathematical model described by Ogihara and Yamazaki (2001), as follows:

(4) $$\begin{array}{l} F_{m}={\bar{F}}_{m}^{\text{CE}}\cdot k\left(\xi_{m}\right)\cdot h\left(\eta_{m}\right)\cdot \alpha_{m}+F_{m}^{\text{PD}}+F_{m}^{\text{PE}}\\ k\left(\xi_{m}\right)=0.32+0.71\exp\left[-1.112\left(\xi_{m}-1\right)\right]\sin\left[3.722\left(\xi_{m}-0.656\right)\right]\\ h\left(\eta_{m}\right)=1+\tanh\left(3.0\eta_{m}\right)\\ F_{m}^{\text{PD}}=c_{m}^{\text{PD}}{\dot{L}}_{m}\\ F_{m}^{\text{PE}}=k_{m}^{\text{PE}}\left\{\exp\left[15\left(L_{m}-{\bar{L}}_{m}\right)\right]-1\right\}, \end{array}$$

where *F*_*m*_ is the muscle tension generated at the *m*th muscle, ${\bar{F}}_{m}^{\text{CE}}$ is the maximum muscle tension by contractile element (CE), *k*(ξ_*m*_) is the force-length relationship, *h*(η_*m*_) is the force-velocity relationship, α_*m*_ is the stimulation signal from the corresponding α-motoneuron (0 ≤ α_*m*_ ≤ 1), $F_{m}^{\text{PD}}$ and $F_{m}^{\text{PE}}$ are the forces generated by the damping and elastic elements. ξ_*m*_ and η_*m*_ are the normalized muscle length and contractile velocity divided by the muscle optimal length ${\bar{L}}_{m}$ and maximum muscle contractile velocity ${\bar{\dot{L}}}_{m}$, that is, where $\xi_{m}=L_{m}/{\bar{L}}_{m}$, $\eta_{m}={\dot{L}}_{m}/{\bar{\dot{L}}}_{m}$, *L*_*m*_, and ${\dot{L}}_{m}$ are the muscle length and the contractile velocity, respectively. $c_{m}^{\text{PD}}$ is the viscous coefficient, and $k_{m}^{\text{PE}}$ is the coefficient of the elastic element.

### 2.3. Nervous System Model

We used Matsuoka neuron model (Matsuoka, 1987) as follows:

(5) $$\begin{array}{l} \begin{array}{c} \tau_{i}\dot{u_{i}}=-u_{i}+\overset{n}{\underset{j=1}{\sum }}w_{ij}^{\text{CPG}}y_{j}-\beta v_{i}+u_{\theta }+{\text{Feed}}_{i},\\ \tau_{i}^{\prime }{\dot{v}}_{i}=-v_{i}+y_{i},\\ y_{i}=\text{max}\left(0,u_{i}\right), \end{array} \end{array}$$

where *u*_*i*_ is the inner state of the *i*th neuron, *v*_*i*_ is a variable that represents the self-inhibition effect of the *i*th neuron. τ_*i*_ and $\tau_{i}^{\prime }$ are time constants, β is a coefficient, *w*_*ij*_ is a connecting weight from the *j*th neuron to the *i*th neuron. *u*_θ_ is an external input with a constant rate, and Feed_*i*_ is a feedback signal from the musculoskeletal system. Parameter values are show in Appendix.

We built a CPG model that is a two-layer hierarchical network composed of a rhythm generator (RG) network and a pattern formation (PF) network based on Li et al. (2013) (Figure 1A). The RG is composed of four Matsuoka neurons mutually inhibited to generate rhythm, whereas, a PF network contains five Matsuoka neurons with mutual and self-inhibition that corresponds to five motor modules. In response to rhythmic activation of the RG as an external input, the five modules are activated one by one sequentially with different phases (Figure 1B). PF neurons issue motor commands to α-motoneurons, which in turn activate muscles. α-motoneurons also receive feedback signals from various reflexes such as posture reflex and crossed extension reflex. α-motoneuron output α_*m*_ and reflex output Reflex_*m*_ are given as follows:

(6) $$\begin{array}{l} \alpha_{m}=w_{m}^{\text{condition}}\frac{1}{1+\exp(-4(\sum_{i=1}^{5}w_{mi}^{\alpha }{\text{PF}}_{i}+{\text{Reflex}}_{m}))},\; \end{array}$$

(7) $$\begin{array}{l} {\text{Reflex}}_{m}=\{\begin{array}{cc} \sum_{j}(c_{mj}\theta_{j}+c_{mj}^{\prime }{\dot{\theta }}_{j})+{\text{POS}}_{m} & \text{GRF}>0,\\ 0 & \text{otherwise,} \end{array} \end{array}$$

where $w_{m}^{\text{condition}}$, $w_{mi}^{\alpha }$, *c*_*mj*_, and $c_{mj}^{\prime }$ are the weight coefficients, PF_*i*_ is the output of PF neuron. θ_*j*_ is the joint angle (*j* ∈ {hip, knee, and ankle} for each leg), POS_*m*_ is a posture control, and GRF is the vertical ground reaction force.

### 2.4. Parameter Search

**Figure 2:**
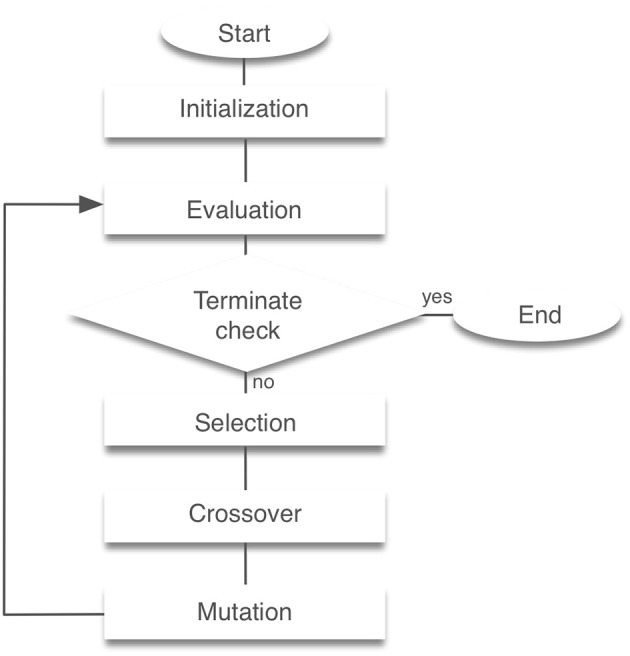
Block diagram of genetic algorithms. Initialization creates a population of random individuals. The evaluation phase tests individuals in the population for fitness. After that, a new generation is created through selection, crossover and mutation. This process is then repeated the required number of times was completed.

Our model has 56 free parameters ($w_{mi}^{\alpha },c_{mj},c_{mj}^{\prime },{\text{POS}}_{m},{\text{Feed}}_{i}$) need to be fixed for stable locomotion. We employed standard genetic algorithms (GAs) for searching these parameters (Noori et al., 2017). GAs are search algorithms based on the biological genetic mechanisms such as selection, crossover, and mutation (Hase and Yamazaki, 2002). The search mechanism is built on the interaction between individuals and the external environment. GAs comprise a set of individuals (the population) and a set of the genetic operators. The individuals have genes that are the potential solutions for the problem. GAs generate a sequence of populations by using genetic operators (selection, crossover, and mutation) among individuals. Individuals that achieved the highest evaluation can survive and generate children. The block diagram of a GA is presented in Figure 2. For the evaluation function, we used the following equation:

(8) $$\begin{array}{l} w_{d}D+w_{s}S\to \text{max} \end{array}$$

where *w*_*d*_ and *w*_*s*_ are the weight coefficients, *D* is the distance until the model falls down, and *S* is the number of steps taken while walking. We initialized 50 individuals randomly. We chose a one-point crossover with a probability of 0.7 and mutation with a probability of 0.1. We carried out impairment simulation for 5 times with 5 different seeds of the random number generator to examine the simulation results are unique. We confirmed that the model achieved stable bipedal locomotion for 5 s, and locomotion patterns with different seeds did not differ qualitatively. Essentially, humans acquire walking by trial and error. We mimicked this using GAs.

### 2.5. Normal and Pathological Locomotion Model

Normal locomotion was simulated by fitting the 56 free parameters by GAs while setting $w_{m}^{\text{condition}}=1.0$ in Equation (3) for any *m*. After the model acquired normal locomotion, we simulated a pathological condition such as stroke. Specifically, we set $w_{m}^{\text{condition}}=0.8$ for *m* = 1, ⋯, 6 and 0.6 for *m* = 7, ⋯, 9 to simulate weakening of MEP signals observed in stroke patients (Piron et al., 2005). Immediately after this manipulation, the model did not succeed to walk even for one step. Then, we examined two “simulated rehabilitation” scenarios for the model. The first scenario is that under the assumption of that the patients could recover the locomotion by compensating their reflexes, we refitted the 28 parameters for reflexes ($c_{mj},c_{mj}^{\prime }$). We call this scenario “reflex-compensation model.” The other scenario is that under the assumption of that the patients restore the locomotion by adapting the controller itself, we refitted the 48 parameters for feedback inputs to the CPG (Feed_*i*_). We call this scenario “CPG-compensation model.” In both scenarios, the model reacquired the locomotion successfully, whereas the motor modules exhibited different dynamics.

### 2.6. Implementation

We implemented GAs using a message passing interface (MPI), which is a library for parallel computing. All programs were written in C language, and the fourth-order Runge-Kutta method was used to solve differential equations numerically. The step-size of time was set as 0.1 ms. In addition, HAT is controlled by both muscles and proportional-derivative control to maintain the model upright. The proportional-derivative control is determined by the angular velocity and the difference of the vertical angle. The angular velocity and angle coefficients are 1.0 × 10^2^ and 1.0 × 10^3^, respectively.

## 3. Results

### 3.1. Acquisition of Normal Locomotion Pattern

**Figure 3:**
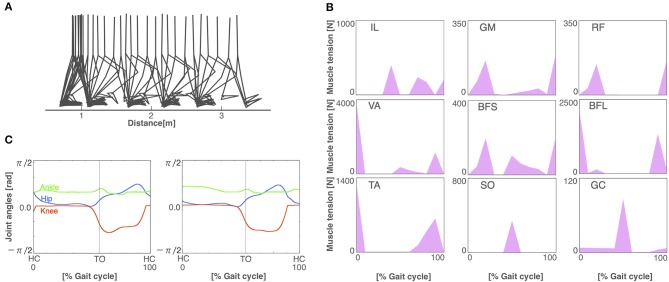
The normal locomotion model. **(A)** Stick diagram of the generated normal locomotion. This is a snapshot of every 0.1 s. **(B)** Muscle tension (vertical axis) of each muscle during the gait cycle (horizontal axis). A gait cycle is the time period of movement during locomotion from the time when the heel of one foot hits the ground to when the heel of that same foot hits the ground again. Abbreviations as in the main text. **(C)** Joint angles in relation to the gait cycle for the left (left panel) and right (right panel) legs. HC and TO indicate the times for heel contact and toe off, respectively.

After 3,000 generations of GAs, the model acquired stable bipedal locomotion, as shown in Figure 3A. The locomotion pattern resembles that of the human biped qualitatively. Figure 3B displays the muscle activations. The iliopsoas (IL) produced the activities in the middle of the gait cycle. The gluteus maximus (GM) and vastus (VA) achieved muscle activity at the beginning and end of the gait cycle. The tibia anterior (TA) obtained the activities at the beginning and middle to the end of the gait cycle. The soleus (SO) and gastrocnemius (GC) produced synchronous activity in the middle of the gait cycle. These muscle activations are consistent with the measured data (Ivanenko et al., 2005). Figure 3C shows the joint kinematics. The waveforms are similar to those previously reported in Ivanenko et al. (2005). The stance phase of the left leg and that of the right leg in gait cycle are 55.6% and 54.5%, respectively, showing that the kinematics of both legs are nearly symmetric. However, several differences between the simulation results and measured data were observed. For example, the RF produced the peak of the muscle activities in the middle of the gait cycle in the measured data. The period of the flexor angle on the knee joint was longer than the measured data.

### 3.2. Walking Pattern of Pathological Condition

**Figure 4:**
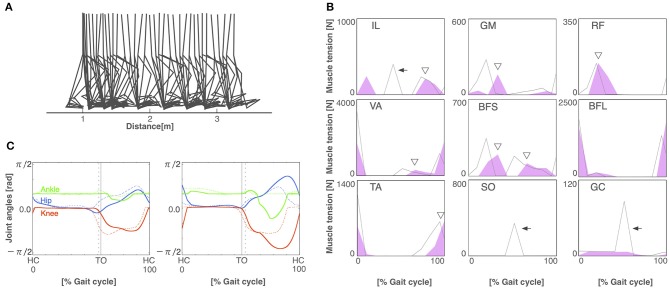
The reflex-compensation model. **(A)** Stick diagram of the generated reflex-compensation locomotion. **(B)** Muscle tension of each muscle during the gait cycle in the reflex-compensation model (red-shaded area) and the normal locomotion model (gray line). Arrows indicate the disappearance of muscle activity. Triangles exhibit that the activities of the muscles shifted later. Abbreviations as in the main text. **(C)** Joint angles in relation to the gait cycle for the left (left panel) and right (right panel) legs in the reflex-compensation model (solid lines) and the normal locomotion model (dashed lines). Conventions as in Figure 3A.

**Figure 5:**
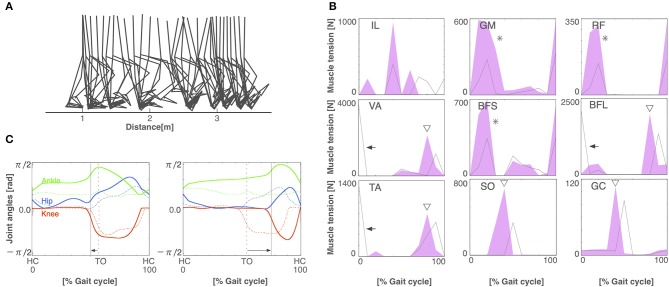
The CPG-compensation model. **(A)** Stick diagram of the generated CPG-compensation locomotion. **(B)** Muscle tension of each muscle during the gait cycle in the CPG-compensation model (red-shaded area) and the normal locomotion model (gray line). Arrows indicate the disappearance of initial muscle activity. Asterisks show that the initial activity of muscles is larger than that in the normal locomotion model. Triangles exhibit that the activities of the muscles shifted earlier. **(C)** Joint angles in relation to the gait cycle for the left (left panel) and right (right panel) legs in the CPG-compensation model (solid lines) and the normal locomotion model (dashed lines). Arrows indicate the shifted stance phase compared to the normal locomotion model. Both legs are asymmetrical throughout the gait cycle. Conventions as in Figure 3A.

To build the pathological locomotion model, we weakened the output neural signal to the muscle on the left leg in the normal locomotion model. The pathological locomotion model fell down immediately when the model attempted to walk. After 200 generations of GA, the reflex-compensation model acquired bipedal locomotion by changing the reflex parameters (Figure 4A). After 1,000 generations of GA, the CPG-compensation model acquired bipedal locomotion by changing the CPG parameters (Figure 5A). In the reflex-compensation model, the step size was smaller than that in the normal locomotion (Figure 4A). In the muscle activations for the affected (left) leg, the activity in IL, SO, and GC observed in the normal locomotion disappeared (Figure 4B). In addition, the activities of the muscles shifted later in the IL, GM, RF, VA, BFS, and TA. The joint angles of the left leg (affected leg) is larger than that of the right leg (unaffected leg) (Figure 4C). The stance phase of the left leg and the right leg in the gait cycle are 60.9% and 50.9%, respectively, suggesting that the locomotor pattern is symmetric. On the other hand, in the CPG-compensation model, the step size varied across gait cycles compared with the normal locomotion (Figure 5A). In other words, the walking pattern of the pathological locomotion model was unstable. In the muscle activations for the affected (left) leg, the initial activity in VA, TA, and BFL observed in the normal locomotion disappeared. In contrast, the initial activity in the GM, RF, and BFS became larger than that in the normal locomotion model. Those muscle activities were generated by the reflex (Equation 7) to compensate for the disappearance of the initial activity in the muscles. In addition, the phases of the activities were advanced in the VA, BFL, TA, SO, and GC (Figure 5B). Related to the phase advances, the stance phase of the left leg (affected leg) became shorter than that of the right leg (unaffected leg) (49.9% and 75.2%, respectively), showing an asymmetric locomotion (Figure 5C).

### 3.3. Comparison of Joint Angles Between Normal and Pathological Walking

**Figure 6:**
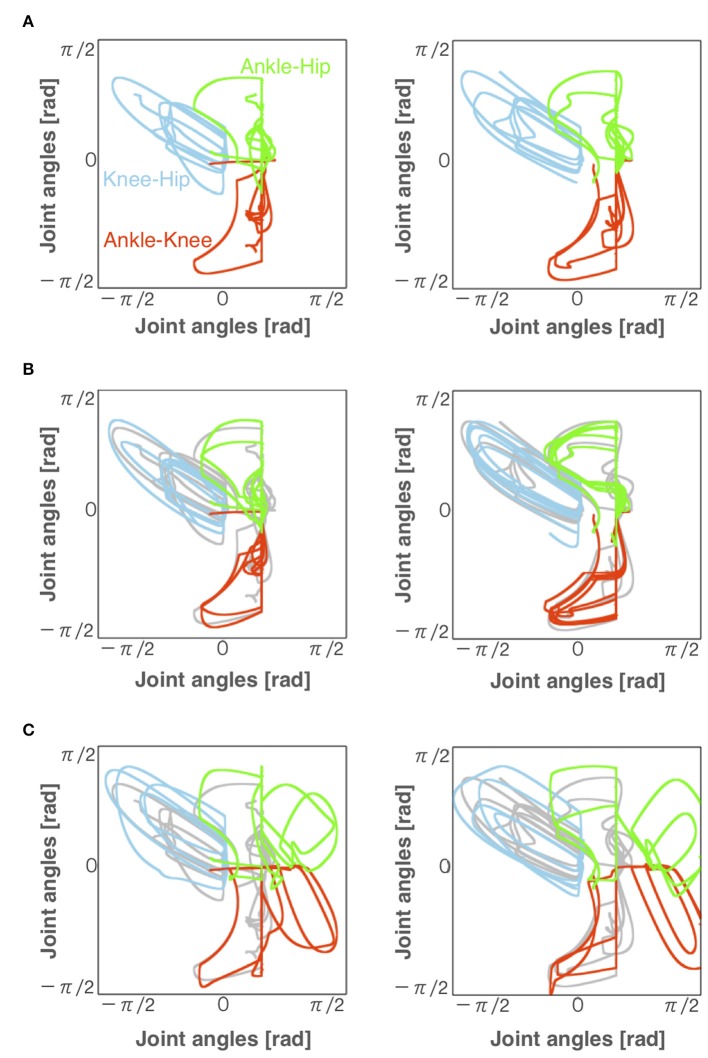
Angles of each joint for left and right legs (left and right panels, respectively) during locomotion in the **(A)** normal, **(B)** reflex-compensation, and **(C)** CPG-compensation models. The horizontal axis and vertical axis are joint angles (radian). Gray lines represents the angles for the normal locomotion model for comparison.

Figure 6 illustrates angle-angle plots to confirm the error of gait trajectories obtained from the normal locomotion model and pathological locomotion models. These figures show that the trajectories for the reflex-compensation model were similar to these for the normal locomotion model (Figure 6B), whereas these for the CPG-compensation model was not (Figure 6C), especially ankle-hip and ankle-knee coordinates. Moreover, these error plots for the CPG-compensation model display that the angles of ankles were not periodic. The unnatural movements imply compensation for acquiring bipedal locomotion. Such a different ankle control strategy seems necessary for stroke patients to walk. In fact, various ankle-foot orthoses have been developed to adjust ankle movements for stroke patients (Ohata et al., 2011). Our simulation result on the CPG-compensation model supports this observation.

### 3.4. Comparison of Motor Modules Between Normal and Pathological Walking

**Figure 7:**
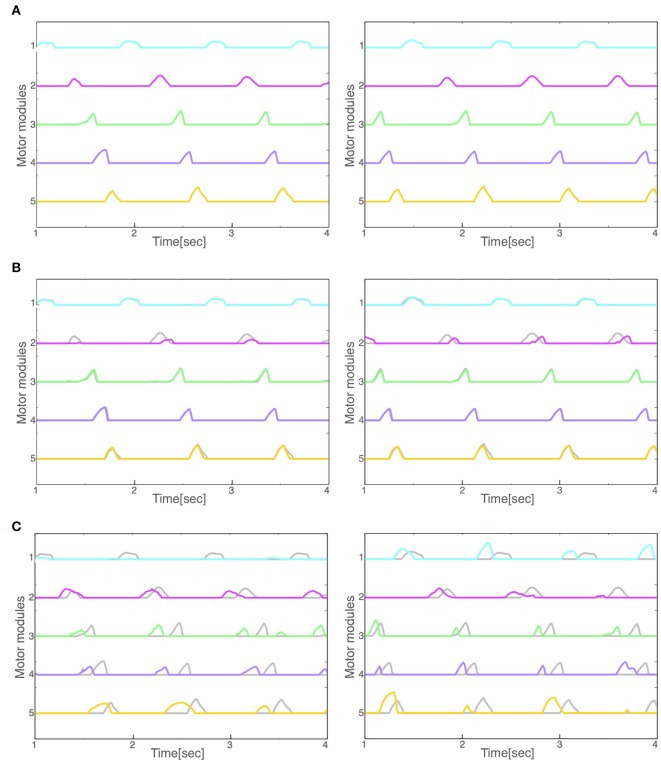
Activity patterns of the motor modules for left and right legs (left and right panels, respectively) during locomotion in the **(A)** normal, **(B)** reflex-compensation, and **(C)** CPG-compensation model. The horizontal axis is time (s) and the vertical axis activity (arbitrary units). Gray lines represents the activity for the corresponding module in the normal model for comparison.

**Figure 8:**
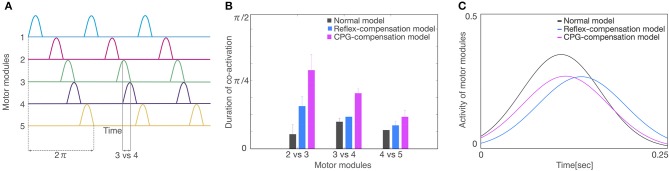
Analysis of activity patterns of the motor modules. **(A)** Schematic representation of activity patterns of the motor modules. One locomotion cycle is an activity period during the 1st to 5th motor modules. The locomotion cycle was set to 2π radian (dashed lines) to compare different locomotion cycles in each model. A duration of co-activation was defined when each module was active at the same time (the duration for modules 3 vs. 4 was shown). **(B)** The average of the co-activation for 3 locomotion cycles in each model. The horizontal axis is a comparison of motor modules and the vertical axis is the duration of co-activation. The colors of gray, blue, and red indicate normal model, reflex-compensation model, and CPG-compensation model, respectively. **(C)** The waveforms by a Gaussian function for the 3 locomotion cycles on the 2nd, 3rd, and 4th motor modules are displayed as normal model (gray), reflex-compensation model (blue), and CPG-compensation model (red).

Finally, we compared the activity patterns of the motor modules, 5 for left and 5 for right legs, in the normal and pathological conditions. Figure 7A illustrates them in the normal condition. All modules exhibit rhythmic activity with different phases; and thus, they become active one by one sequentially. In particular, the offset of the activity in the 5th module (the end of a locomotion cycle) is followed by the onset in the 1st module (the start of the next cycle) continuously. Figure 7B shows them in the reflex-compensation model. In this case, all the modules exhibited activities one by one sequentially as in the normal condition, although the phase of the 2nd module in both legs was delayed than that in the normal condition. Figure 7C shows the activity patterns in the CPG-compensation model. In this case, the 1st module for the affected (left) leg did not show marked activity. Moreover, in the 3rd, 4th, and 5th modules, the phases were advanced. We analyzed how long 2 motor modules were activated simultaneously, and plotted the durations between the 2nd and 3rd modules, those between the 3rd and 4th modules, and those between the 4th and 5th modules (Figure 8). The duration in the CPG-compensation model was the longest, whereas these were comparable in the Reflex-compensation model and the normal model (Figure 8B), suggesting that the activations of motor modules in the CPG-compensation model overlap temporally as if they are merged as a single module (Figure 8B). However, the overlap might be caused by a prolonged activity of each motor module. To address this, we analyzed the duration of 2nd, 3rd, and 4th modules for each condition by fitting the activities of the modules by a Gaussian function (Figure 8C). The Full-width at half maximum (FWHM) of the waveforms in the normal model, reflex-compensation model, and CPG-compensation model were 12.467, 13.730, and 13.528, respectively. Thus, the motor modules in the CPG-compensation model showed the same duration with those in the normal model, suggesting that the active durations are not extended. These results conclude that in the CPG-compensation model, motor modules are co-activated in time more than those in the normal and Reflex-compensation models, as if the modules act as a “merged” single module. The merging of motor modules is also observed in the unaffected leg, suggesting that the deficit in one leg affects the other leg.

## 4. Discussion

**Figure 9:**
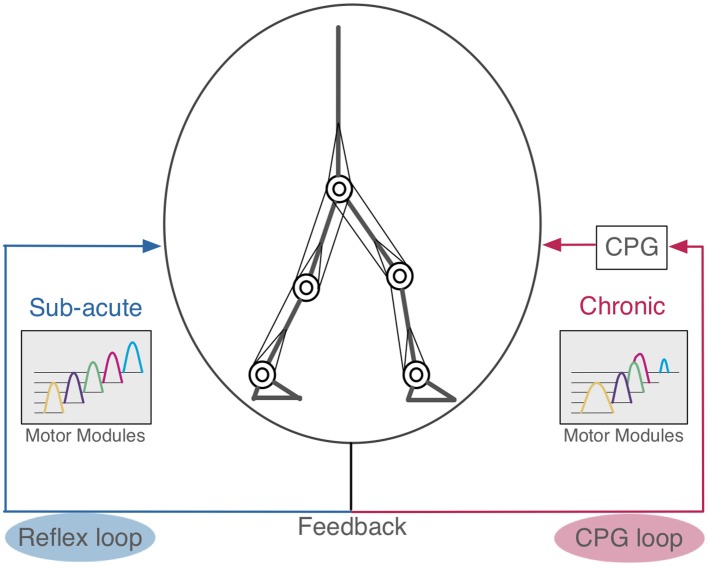
Hypothetical scheme of compensation in human locomotion. The left side is the reflex feedback loop. When the pathological patients acquired bipedal locomotion by modifying the reflex loop, their motor modules were not merged similar to sub-acute stroke patients (Gizzi et al., 2011). The right side is the CPG feedback loop. If these motor modules were merged, this suggests the patients that had changed their CPG feedback loop to acquire bipedal locomotion, as observed in chronic stroke patients (Clark et al., 2010).

In this study, we carried out computer simulation of bipedal locomotion in normal and pathological conditions. In normal condition, our neuromuscloskeletal model, composed of a lower body with 7 links and 18 muscles, and a CPG controller with RG and PF organized hierarchically, walked successfully after fitting the internal parameters through a GA algorithm. In pathological conditions, input signals from the controller to one side of the leg were weakened, according to experimental results (Piron et al., 2005). This manipulation led to the immediate failure of stable walking. Then, we examined two scenarios to recover the stable walking. In one situation, parameters for proprioceptive feedback inputs were refitted, which simulates patients compensating locomotion by adapting reflexes against proprioceptive feedback signals. In this scenario, the model successfully acquired the stable walking again, and the activity patterns of motor modules were unaffected against the manipulation, although there were some marked differences in muscle activity patterns. In the other scenario, in which parameters for the CPG were refitted to simulate patients compensating the locomotion by adapting the controller, phases were advanced across multiple modules, and the modules tended to became active with identical phases, as if they were “merged” as a single module. These results suggest that if the compensation was made by adapting the reflex against feedback signals as light on sub-acute stroke patients would do, the motor modules were unaffected, whereas if the compensation was made by adapting the controller as severe on chronic patients would do, the modules were merged (Figure 9). These observations are consistent with experimental findings (Clark et al., 2010; Gizzi et al., 2011).

### 4.1. Normal Locomotion Model

The model is built based on a musculoskeletal model (Taga et al., 1991; Taga, 1995; Ogihara and Yamazaki, 2001) and a CPG model (Jo and Massaquoi, 2007; Aoi et al., 2010, 2012). The resulting model contains 56 free parameters to be fitted to acquire stable walking. Previous studies have fitted these parameters by hand (Jo and Massaquoi, 2007), reinforcement learning (Matsubara et al., 2006; Li et al., 2013), and GAs (Ogihara and Yamazaki, 2001; Hase and Yamazaki, 2002). Notably, Aoi et al. (2019) compared gait patterns of healthy subjects with a computer simulation model with a CPG controller, where the 69 parameters were hand-tuned. We employed a GA algorithm for fitting the parameters that maximizes the walking distance and the number of steps. The GA succeeded to find the appropriate values for the 56 parameters. Eventually, our model acquired a symmetrical locomotion pattern (Figure 3A), which is consistent with normal human locomotion pattern (Ivanenko et al., 2005).

### 4.2. Pathological Locomotion Model

We then examined two possible scenarios for stroke patients to recover stable locomotion, reflex-compensation and CPG-compensation, where the former assumes that patients modify the reflexes while the latter they modify controller. We observed that in the former, the motor modules were not merged, whereas in the latter they were. Where does the different come from? Allen et al. (2013) built a pathological gait model based on the stroke patient data (Clark et al., 2010), and analyzed how different module patterns affect the locomotion. Specifically, they demonstrated that different merge combinations of motor modules results in difference locomotion impairments. However, they did not consider how such merging of motor modules would occur in stroke patients. On the other hand, we observed that CPG-compensation model showed the merge of motor modules to reacquire stable locomotion, suggesting that the merge occurs internally through the reacquisition process of locomotion. Meanwhile, we also observed that our reflex-compensation model did not merge the motor modules. This results seems consistent with the experimental findings by Gizzi et al. (2011), showing that sub-acute stroke patients did not show the merge of motor modules. These results suggest that depending on the duration after stroke, the patients would take different strategies to compensate their locomotion behaviors through the reacquisition process. The spinal cord network has the functions of adaptation by the feedback signals (Rossignol et al., 2006). In stroke patients, spinal cord is intact in general. For this reason, we propose that when the post-stroke period is short, the reflex feedback loop is affected, whereas when the duration is long, the CPG feedback loop is affected (Figure 9).

### 4.3. Implications for Adaptive Robot Locomotion

CPG-based bipedal robots change their locomotion patterns adaptively against environmental changes to some extent owing to the entrainment of the dynamical systems. For example, CPG-based robots can keep walking on a flat ground as well as a slope (Taga et al., 1991; Taga, 1995; Ishiguro et al., 2003; Matsubara et al., 2006; Aoi et al., 2010; Li et al., 2013, 2017) by sensing the acceleration change provided by proprioceptive feedback inputs to the controller. However, this ability requires an intact musculoskeletal system. When the system malfunctions, stable locomotion pattern would not be expected anymore. In this study, we demonstrated that even if the system malfunctions, as simulated stroke conditions, the system could recover the stable locomotion again by modifying internal parameters. However, depending on the choice of parameters to update, additional parameter search takes longer relearning time, and the strategy for restoring the locomotion patterns differs. In particular, motor modules are merged so that robots would exhibit only poor locomotion patterns in a certain condition. These results suggest that not all parameters should be updated to restore the locomotion ability.

### 4.4. Impact on Online Rehabilitation

Previous studies have shown that online rehabilitation has potentially useful attributes (Velliste et al., 2008). Therefore, various methods have been suggested for detecting motions of patients easily and accurately (Naseer et al., 2014; Holtzer et al., 2015; Khan et al., 2018). In particular, Khan et al. (2018) proposed a novel methodology that controls a prosthetic leg computing model using functional near-infrared spectroscopy (fNIRS) signals with little errors. This result can be effectively used for the rehabilitation of lower-limb amputees and patients with paralysis. This system currently supports only a few commands to generate motor torques to move forward or backward because of a crude resolution of fNIRS signals. In spite of this, more commands will be desirable. The fNIRS signals are measured during locomotion on a treadmill, and so the signals would reflect activities of CPGs within the brain. We could use our CPG model as a model of the generator of the fNIRS signals to enhance the signal resolution.

### 4.5. Limitations of This Study

Our musculoskeletal model is restricted in 2D, and the CPG model is abstracted mathematically. Thus, the model would not be suitable to study more detailed locomotion movements in a 3D space, as demonstrated by Kim et al. (2011) for example, or neuronal activity during locomotion in the brain. Rather, our model provides a crude closed-loop system model for human locomotion that can be somehow manipulated to simulate pathological situation. We will release the source code of our model under a opensource license to let researchers to elaborate the present model in future.

### 4.6. Suggestions for Rehabilitation Therapy

Hashiguchi et al. (2016) reported that the reduced number of motor modules in stroke patients was recovered to the original level by the rehabilitation for one month, and the tendency of the restoration was correlated with the improvement of muscle strengths and gait patterns by the rehabilitation. When the muscle strengths were weak, the patients would be impossible to compensate locomotion only by the reflexes, and so would need to change the motor strategy by changing the CPGs itself. This observation seems consistent with the present study, because compensation by the reflex did not affect motor modules, whereas that by the CPG did. Robot assisted locomotor training is a useful means for treatment of impaired locomotion (Jezernik et al., 2003; Kawamoto et al., 2013; Molteni et al., 2017; Watanabe et al., 2017) and the devices have been drastically improved to date (Beckerle et al., 2017; Gandolla et al., 2018; Mummolo et al., 2018). Tan et al. (2018) reported that robotic-assisted locomotor training for stroke patients improves walking speed, step cadence, stance duration percentage of gait cycle, but does not increase the number of motor modules. This implies that if more motor modules are recruited, the realized locomotion will be more sophisticated. Summarizing, stroke patients should be engaged with muscle exercises as well as gait training so that they could compensate the locomotion only by the reflex.

## 5. Conclusion

In this study, we investigated what conditions in stroke patients would result in the merge of motor modules. We built a musculoskeletal bipedal locomotion model with a neural controller. In a simulated stroke condition, the motor modules in the controller were merged functionally, suggesting that chronic stroke patients would modify internal parameters for the controller to recover locomotion. These results would thus provide insights on how the motor modules are merged in stroke patients for better rehabilitation. These findings also help to provide a means for robots to recover locomotion adaptively after malfunctioning of the controller.

## Data Availability Statement

All datasets generated for this study are included in the manuscript/Supplementary Files.

## Author Contributions

DI and TY designed the research, analyzed data, and wrote the paper. DI performed the research.

### Conflict of Interest

The authors declare that the research was conducted in the absence of any commercial or financial relationships that could be construed as a potential conflict of interest.
