# Supplementary material for: A Pathological Condition Affects Motor Modules in a Bipedal Locomotion Model
Source: Front Neurorobot. 2019 Sep 20;13:79. doi: 10.3389/fnbot.2019.00079 (PMC6763684; doi:10.3389/fnbot.2019.00079)
Supplement: Supplementary file 1 [file Data_Sheet_1.pdf]

Appendix:
A pathological condition affects motor modules in a bipedal locomotion model

A.1 Free parameters determined by GA

Free parameters determined by a GA are as follows:

(a) Normal locomotion model

$G_1^{\mathrm{RG}} = -1.200$, $G_2^{\mathrm{RG}} = 0.080$, $G_3^{\mathrm{RG}} = 0.010$, $G_4^{\mathrm{RG}} = 0.010$, $G_5^{\mathrm{RG}} = 0.010$, $G_6^{\mathrm{RG}} = -0.010$,
$G_7^{\mathrm{RG}} = G_1^{\mathrm{RG}}$, $G_8^{\mathrm{RG}} = G_2^{\mathrm{RG}}$, $G_9^{\mathrm{RG}} = G_3^{\mathrm{RG}}$, $G_{10}^{\mathrm{RG}} = G_4^{\mathrm{RG}}$, $G_{11}^{\mathrm{RG}} = G_5^{\mathrm{RG}}$, $G_{12}^{\mathrm{RG}} = G_6^{\mathrm{RG}}$,
$G_1^{\mathrm{PF}} = -0.198$, $G_2^{\mathrm{PF}} = 0.016$, $G_3^{\mathrm{PF}} = 0.057$, $G_4^{\mathrm{PF}} = 0.010$, $G_5^{\mathrm{PF}} = -0.022$, $G_6^{\mathrm{PF}} = 0.710$, $G_7^{\mathrm{PF}} = -0.010$,
$G_8^{\mathrm{PF}} = 0.510$, $G_9^{\mathrm{PF}} = -0.198$, $G_{10}^{\mathrm{PF}} = 0.198$, $G_{11}^{\mathrm{PF}} = -0.058$, $G_{12}^{\mathrm{PF}} = -0.013$, $G_{13}^{\mathrm{PF}} = 0.010$, $G_{14}^{\mathrm{PF}} = 0.000$,
$G_{15}^{\mathrm{PF}} = 0.085$, $G_{16}^{\mathrm{PF}} = 0.012$, $G_{17}^{\mathrm{PF}} = 0.104$, $G_{18}^{\mathrm{PF}} = -0.010$,
$G_{19}^{\mathrm{PF}} = G_1^{\mathrm{PF}}$, $G_{20}^{\mathrm{PF}} = G_2^{\mathrm{PF}}$, $G_{21}^{\mathrm{PF}} = G_3^{\mathrm{PF}}$, $G_{22}^{\mathrm{PF}} = G_4^{\mathrm{PF}}$, $G_{23}^{\mathrm{PF}} = G_5^{\mathrm{PF}}$, $G_{24}^{\mathrm{PF}} = G_6^{\mathrm{PF}}$, $G_{25}^{\mathrm{PF}} = G_7^{\mathrm{PF}}$, $G_{26}^{\mathrm{PF}} = G_8^{\mathrm{PF}}$,
$G_{27}^{\mathrm{PF}} = G_9^{\mathrm{PF}}$, $G_{28}^{\mathrm{PF}} = G_{10}^{\mathrm{PF}}$, $G_{29}^{\mathrm{PF}} = G_{11}^{\mathrm{PF}}$, $G_{30}^{\mathrm{PF}} = G_{12}^{\mathrm{PF}}$, $G_{31}^{\mathrm{PF}} = G_{13}^{\mathrm{PF}}$, $G_{32}^{\mathrm{PF}} = G_{14}^{\mathrm{PF}}$, $G_{33}^{\mathrm{PF}} = G_{15}^{\mathrm{PF}}$, $G_{34}^{\mathrm{PF}} = G_{16}^{\mathrm{PF}}$,
$G_{35}^{\mathrm{PF}} = G_{17}^{\mathrm{PF}}$, $G_{36}^{\mathrm{PF}} = G_{18}^{\mathrm{PF}}$,
$G_1^{\alpha} = 0.650$, $G_2^{\alpha} = -0.278$, $G_3^{\alpha} = 0.499$, $G_4^{\alpha} = 0.456$, $G_5^{\alpha} = 0.139$, $G_6^{\alpha} = 0.919$, $G_7^{\alpha} = 0.215$,
$G_8^{\alpha} = -0.027$, $G_9^{\alpha} = 1.001$, $G_{10}^{\alpha} = 0.050$, $G_{11}^{\alpha} = -0.144$, $G_{12}^{\alpha} = -0.023$, $G_{13}^{\alpha} = 0.401$, $G_{14}^{\alpha} = 0.178$,
$G_{15}^{\alpha} = 0.323$, $G_{16}^{\alpha} = 0.014$, $G_{17}^{\alpha} = 0.240$, $G_{18}^{\alpha} = 0.715$,
$G_1^{c} = 0.428$, $G_2^{c} = 0.219$, $G_3^{c} = 0.128$, $G_4^{c} = 0.030$, $G_5^{c} = 0.128$, $G_6^{c} = -0.049$, $G_7^{c} = 0.202$, $G_8^{c} = G_1^{c}$,
$G_9^{c} = G_2^{c}$, $G_{10}^{c} = G_3^{c}$, $G_{11}^{c} = G_4^{c}$, $G_{12}^{c} = G_5^{c}$, $G_{13}^{c} = G_6^{c}$, $G_{14}^{c} = G_7^{c}$, $G_1^{c'} = 0.432$, $G_2^{c'} = 0.351$,
$G_3^{c'} = G_1^{c'}$, $G_4^{c'} = G_2^{c'}$,
$G_1^{\mathrm{POS}} = 0.208$, $G_2^{\mathrm{POS}} = 0.390$, $G_3^{\mathrm{POS}} = 0.525$, $G_4^{\mathrm{POS}} = 0.212$, $G_5^{\mathrm{POS}} = -0.191$.
$G_6^{\mathrm{POS}} = G_1^{\mathrm{POS}}$, $G_7^{\mathrm{POS}} = G_2^{\mathrm{POS}}$, $G_8^{\mathrm{POS}} = G_3^{\mathrm{POS}}$, $G_9^{\mathrm{POS}} = G_4^{\mathrm{POS}}$, $G_1^{\mathrm{POS}}0 = G_5^{\mathrm{POS}}$.

(b) Pathological locomotion model: reflex-compensation model

$G_1^{c} = 0.453$, $G_2^{c} = 0.087$, $G_3^{c} = 0.937$, $G_4^{c} = 0.800$, $G_5^{c} = 0.976$, $G_6^{c} = 0.225$, $G_7^{c} = -0.135$,
$G_8^{c} = 0.348$, $G_9^{c} = 0.112$, $G_{10}^{c} = 0.925$, $G_{11}^{c} = 0.847$, $G_{12}^{c} = 1.042$, $G_{13}^{c} = 0.193$, $G_{14}^{c} = -0.136$,
$G_1^{c'} = 0.737$, $G_2^{c'} = 0.331$,
$G_3^{c'} = 0.837$, $G_4^{c'} = 0.403$,
$G_1^{\mathrm{POS}} = 0.471$, $G_2^{\mathrm{POS}} = 0.206$, $G_3^{\mathrm{POS}} = 0.424$, $G_4^{\mathrm{POS}} = 0.172$, $G_5^{\mathrm{POS}} = -0.160$.
$G_6^{\mathrm{POS}} = 0.386$, $G_7^{\mathrm{POS}} = 0.192$, $G_8^{\mathrm{POS}} = 0.453$, $G_9^{\mathrm{POS}} = 0.420$, $G_{10}^{\mathrm{POS}} = -0.162$.
Other parameters were used by the normal locomotion model parameters.

(c) Pathological locomotion model: CPG-compensation model

$G_1^{\mathrm{RG}} = -9.270$, $G_2^{\mathrm{RG}} = 0.293$, $G_3^{\mathrm{RG}} = 0.874$, $G_4^{\mathrm{RG}} = 0.854$, $G_5^{\mathrm{RG}} = 0.633$, $G_6^{\mathrm{RG}} = 0.151$,
$G_7^{\mathrm{RG}} = -0.276$, $G_8^{\mathrm{RG}} = 1.749$, $G_9^{\mathrm{RG}} = 1.067$, $G_{10}^{\mathrm{RG}} = 0.841$, $G_{11}^{\mathrm{RG}} = 0.359$, $G_{12}^{\mathrm{RG}} = 0.664$,

$G_1^{\mathrm{PF}} = -0.101$, $G_2^{\mathrm{PF}} = 0.001$, $G_3^{\mathrm{PF}} = 0.030$, $G_4^{\mathrm{PF}} = 0.010$, $G_5^{\mathrm{PF}} = -0.001$, $G_6^{\mathrm{PF}} = 0.020$, $G_7^{\mathrm{PF}} = -0.001$, $G_8^{\mathrm{PF}} = 0.001$, $G_9^{\mathrm{PF}} = -0.020$, $G_{10}^{\mathrm{PF}} = 0.012$, $G_{11}^{\mathrm{PF}} = -0.010$, $G_{12}^{\mathrm{PF}} = -0.004$, $G_{13}^{\mathrm{PF}} = 0.055$, $G_{14}^{\mathrm{PF}} = 0.001$, $G_{15}^{\mathrm{PF}} = 0.001$, $G_{16}^{\mathrm{PF}} = 0.001$, $G_{17}^{\mathrm{PF}} = 0.003$, $G_{18}^{\mathrm{PF}} = -0.057$,
$G_{19}^{\mathrm{PF}} = -0.001$, $G_{20}^{\mathrm{PF}} = 0.001$, $G_{21}^{\mathrm{PF}} = 0.002$, $G_{22}^{\mathrm{PF}} = 0.050$, $G_{23}^{\mathrm{PF}} = -0.002$, $G_{24}^{\mathrm{PF}} = 0.001$, $G_{25}^{\mathrm{PF}} = -0.003$, $G_{26}^{\mathrm{PF}} = 0.020$, $G_{27}^{\mathrm{PF}} = -0.001$, $G_{28}^{\mathrm{PF}} = 0.007$, $G_{29}^{\mathrm{PF}} = -0.007$, $G_{30}^{\mathrm{PF}} = -0.001$, $G_{31}^{\mathrm{PF}} = 0.014$, $G_{32}^{\mathrm{PF}} = 0.001$, $G_{33}^{\mathrm{PF}} = 0.002$, $G_{34}^{\mathrm{PF}} = 0.011$, $G_{35}^{\mathrm{PF}} = 0.004$, $G_{36}^{\mathrm{PF}} = -0.010$,
Other parameters were used by the normal locomotion model parameters.

A.2 CPG model parameters

(a) Parameters for RG network

$\tau_1^{\mathrm{RG}}, \cdots, \tau_4^{\mathrm{RG}} = 0.20$, $\tau_1'^{\mathrm{RG}}, \cdots, \tau_4'^{\mathrm{RG}} = 0.50$, $\beta_1^{\mathrm{RG}}, \cdots, \beta_4^{\mathrm{RG}} = 2.00$, $u_{\theta 1}^{\mathrm{RG}}, \cdots, u_{\theta 4}^{\mathrm{RG}} = 2.00$,

$$w_{i\,j}^{\mathrm{CPGRG}} = \begin{cases} -2.0 & (i,j) \in \{(1,3),(1,4),(2,1),(2,4), \\ & \qquad (3,1),(3,2),(4,2),(4,3), \\ 0.0 & \text{otherwise}, \end{cases}$$

Feedback signals from the musculoskeletal system to RG network are given as follows:

$$\begin{aligned}
\mathrm{Feed}_1^{\mathrm{RG}} &= \delta(G_1^{\mathrm{RG}}\mathrm{GRF}_{rh}) + f(G_2^{\mathrm{RG}}, \mathrm{GRF}_{rh}, 0.0) + G_3^{\mathrm{RG}}\theta_{\mathrm{lhip}}, \\
\mathrm{Feed}_2^{\mathrm{RG}} &= f(G_4^{\mathrm{RG}}, \mathrm{GRF}_{lh}, 0.0) + G_5^{\mathrm{RG}}\theta_{\mathrm{rhip}} - G_6^{\mathrm{RG}}\theta_{\mathrm{lhip}}, \\
\mathrm{Feed}_3^{\mathrm{RG}} &= \delta(G_7^{\mathrm{RG}}\mathrm{GRF}_{lh}) + f(G_8^{\mathrm{RG}}, \mathrm{GRF}_{lh}, 0.0) + G_9^{\mathrm{RG}}\theta_{\mathrm{rhip}}, \\
\mathrm{Feed}_4^{\mathrm{RG}} &= f(G_{10}^{\mathrm{RG}}, \mathrm{GRF}_{rh}, 0.0) + G_{11}^{\mathrm{RG}}\theta_{\mathrm{lhip}} - G_{12}^{\mathrm{RG}}\theta_{\mathrm{rhip}}, \\
f(a,b,c) &= \begin{cases} a & \{b > c\}, \\ 0.0 & \text{otherwise}, \end{cases}
\end{aligned}$$

where $\delta()$ is Diac's delta function. GRF_{rh} and GRF_{lh} indicate the ground reaction force from the right heel and the left heel, respectively. θ_{rhip} and θ_{lhip} are the right hip angle and the left hip angle, respectively.

(b) Parameters for PF network

$\tau_1^{\mathrm{PF}}, \cdots, \tau_{10}^{\mathrm{PF}} = 0.08$, $\tau_1'^{\mathrm{PF}}, \cdots, \tau_{10}'^{\mathrm{PF}} = 0.60$, $\beta_1^{\mathrm{PF}}, \beta_6^{\mathrm{PF}} = 15.0$, $\beta_2^{\mathrm{PF}}, \beta_7^{\mathrm{PF}} = 10.0$, $\beta_3^{\mathrm{PF}}, \cdots, \beta_5^{\mathrm{PF}} = 5.00$, $\beta_8^{\mathrm{PF}}, \cdots, \beta_{10}^{\mathrm{PF}} = 5.00$,

$$w_{i\,j}^{\mathrm{CPGPF}} = \begin{cases} -10.0 & (i,j) \in \{(1,2),(2,3),(3,4),(4,5), \\ & \qquad (5,1),(3,5),(6,7),(7,8), \\ & \qquad (8,9),(9,10),(10,6), \\ & \qquad (8,10)\}, \\ -3.0 & (i,j) \in \{(5,8),(10,3),(1,9),(6,4),\}, \\ 1.0 & (i,j) \in \{(1,8),(6,3),(8,1),(3,6), \\ & \qquad (5,7),(7,5),(10,2),(2,10)\}, \\ 0.0 & \text{otherwise}. \end{cases}$$

$$
\begin{aligned}
u_{\theta_1} &= g(-u_4^{\mathrm{RG}}, u_4^{\mathrm{RG}}, -0.30),\\
u_{\theta_2} &= f(0.7u_4^{\mathrm{RG}}, u_4^{\mathrm{RG}}, 0.50),\\
u_{\theta_3} &= f(1.8u_3^{\mathrm{RG}}, u_3^{\mathrm{RG}}, 0.00),\\
u_{\theta_4} &= f(u_3^{\mathrm{RG}}, u_3^{\mathrm{RG}}, 0.50),\\
u_{\theta_5} &= f(u_3^{\mathrm{RG}}, u_3^{\mathrm{RG}}, 0.90),\\
u_{\theta_6} &= g(-u_2^{\mathrm{RG}}, u_2^{\mathrm{RG}}, -0.30),\\
u_{\theta_7} &= f(0.7u_2^{\mathrm{RG}}, u_2^{\mathrm{RG}}, 0.50),\\
u_{\theta_8} &= f(1.8u_1^{\mathrm{RG}}, u_1^{\mathrm{RG}}, 0.00),\\
u_{\theta_9} &= f(u_1^{\mathrm{RG}}, u_1^{\mathrm{RG}}, 0.50),\\
u_{\theta_{10}} &= f(u_1^{\mathrm{RG}}, u_1^{\mathrm{RG}}, 0.90),\\
g(a,b,c) &= \begin{cases} a & \{b < c\}, \\ 0.0 & \text{otherwise.} \end{cases}
\end{aligned}
$$

Feedback signals from the musculoskeletal system to PF network are given as follows:

$$
\begin{aligned}
\mathrm{Feed}_1^{\mathrm{PF}} &= -G_1^{\mathrm{PF}}\theta_{\mathrm{rhip}} + G_2^{\mathrm{PF}}\theta_{\mathrm{lhip}} + G_3^{\mathrm{PF}}\theta_{\mathrm{rknee}}h(\mathrm{GRF}_{rh}) + G_4^{\mathrm{PF}}h(\mathrm{GRF}_{rt}),\\
\mathrm{Feed}_2^{\mathrm{PF}} &= -G_5^{\mathrm{PF}}\theta_{\mathrm{rknee}}h(\mathrm{GRF}_{rh}) + G_6^{\mathrm{PF}}\theta_{\mathrm{rhip}}h(\mathrm{GRF}_{rh}) - G_7^{\mathrm{PF}}\dot{\theta}_{\mathrm{rknee}}h(\mathrm{GRF}_{rh}) + G_8^{\mathrm{PF}}\theta_{\mathrm{rankle}}h(\mathrm{GRF}_{rh}),\\
\mathrm{Feed}_3^{\mathrm{PF}} &= -G_9^{\mathrm{PF}}\theta_{\mathrm{rhip}} + G_{10}^{\mathrm{PF}}\theta_{\mathrm{lhip}} - G_{11}^{\mathrm{PF}}\theta_{\mathrm{rknee}}h(\mathrm{GRF}_{rt}) - G_{12}^{\mathrm{PF}}h(\mathrm{GRF}_{lt}) + G_{13}^{\mathrm{PF}}\theta_{\mathrm{rankle}}h(\mathrm{GRF}_{rh}),\\
\mathrm{Feed}_4^{\mathrm{PF}} &= f(G_{14}^{\mathrm{PF}}, \mathrm{GRF}_{lh}, 0.0),\\
\mathrm{Feed}_5^{\mathrm{PF}} &= G_{15}^{\mathrm{PF}}\theta_{\mathrm{lhip}} + G_{16}^{\mathrm{PF}}h(\mathrm{GRF}_{lt}) + G_{17}^{\mathrm{PF}}\theta_{\mathrm{rankle}}h(\mathrm{GRF}_{rh}) - G_{18}^{\mathrm{PF}}\theta_{\mathrm{rhip}},\\
\mathrm{Feed}_6^{\mathrm{PF}} &= -G_{19}^{\mathrm{PF}}\theta_{\mathrm{lhip}} + G_{20}^{\mathrm{PF}}\theta_{\mathrm{rhip}} + G_{21}^{\mathrm{PF}}\theta_{\mathrm{lknee}}h(\mathrm{GRF}_{lh}) + G_{22}^{\mathrm{PF}}h(\mathrm{GRF}_{lt}),\\
\mathrm{Feed}_7^{\mathrm{PF}} &= -G_{23}^{\mathrm{PF}}\theta_{\mathrm{lknee}}h(\mathrm{GRF}_{lh}) + G_{24}^{\mathrm{PF}}\theta_{\mathrm{lhip}}h(\mathrm{GRF}_{lh}) - G_{25}^{\mathrm{PF}}\dot{\theta}_{\mathrm{lknee}}h(\mathrm{GRF}_{lh}) + G_{26}^{\mathrm{PF}}\theta_{\mathrm{lankle}}h(\mathrm{GRF}_{lh}),\\
\mathrm{Feed}_8^{\mathrm{PF}} &= -G_{27}^{\mathrm{PF}}\theta_{\mathrm{lhip}} + G_{28}^{\mathrm{PF}}\theta_{\mathrm{rhip}} - G_{29}^{\mathrm{PF}}\theta_{\mathrm{lknee}}h(\mathrm{GRF}_{lt}) - G_{30}^{\mathrm{PF}}h(\mathrm{GRF}_{rt}) + G_{31}^{\mathrm{PF}}\theta_{\mathrm{lankle}}h(\mathrm{GRF}_{lh}),\\
\mathrm{Feed}_9^{\mathrm{PF}} &= f(G_{32}^{\mathrm{PF}}, \mathrm{GRF}_{rh}, 0.0),\\
\mathrm{Feed}_{10}^{\mathrm{PF}} &= G_{33}^{\mathrm{PF}}\theta_{\mathrm{rhip}} + G_{34}^{\mathrm{PF}}h(\mathrm{GRF}_{rt}) + G_{35}^{\mathrm{PF}}\theta_{\mathrm{lankle}}h(\mathrm{GRF}_{lh}) - G_{36}^{\mathrm{PF}}\theta_{\mathrm{lhip}},\\
h(a) &= \begin{cases} 1.0 & \{a > 0.0\}, \\ 0.0 & \text{otherwise.} \end{cases}
\end{aligned}
$$

where $\theta_{\mathrm{rknee}}, \theta_{\mathrm{lknee}}, \theta_{\mathrm{rankle}}, \theta_{\mathrm{lankle}}$ are the right knee angle, left knee angle, the right ankle angle, and the left ankle angle, respectively. GRF_{rt} and GRF_{lt}, indicate the ground reaction force from right toe and left toe, respectively.

A.3 α-motoneuron parameters

(a) Normal locomotion model

$w_1^{\mathrm{condition}}, \cdots, w_{18}^{\mathrm{condition}} = 1.0,$
$w_{1\,1}^{\alpha} = G_1^{\alpha},\ w_{1\,5}^{\alpha} = G_2^{\alpha},\ w_{2\,3}^{\alpha} = G_3^{\alpha},\ w_{2\,4}^{\alpha} = G_4^{\alpha},\ w_{3\,1}^{\alpha} = G_5^{\alpha},\ w_{3\,5}^{\alpha} = G_6^{\alpha},\ w_{4\,1}^{\alpha} = G_7^{\alpha},\ w_{4\,3}^{\alpha} = G_8^{\alpha},$

$w^{\alpha}_{5\,1} = G^{\alpha}_{9}$, $w^{\alpha}_{5\,4} = G^{\alpha}_{10}$, $w^{\alpha}_{5\,5} = G^{\alpha}_{11}$, $w^{\alpha}_{6\,1} = G^{\alpha}_{12}$, $w^{\alpha}_{6\,5} = G^{\alpha}_{13}$, $w^{\alpha}_{7\,2} = G^{\alpha}_{14}$, $w^{\alpha}_{8\,2} = G^{\alpha}_{15}$, $w^{\alpha}_{9\,1} = G^{\alpha}_{16}$, $w^{\alpha}_{9\,4} = G^{\alpha}_{17}$, $w^{\alpha}_{9\,5} = G^{\alpha}_{18}$,
$w^{\alpha}_{10\,1} = G^{\alpha}_{1}$, $w^{\alpha}_{10\,5} = G^{\alpha}_{2}$, $w^{\alpha}_{11\,3} = G^{\alpha}_{3}$, $w^{\alpha}_{11\,4} = G^{\alpha}_{4}$, $w^{\alpha}_{12\,1} = G^{\alpha}_{5}$, $w^{\alpha}_{12\,5} = G^{\alpha}_{6}$, $w^{\alpha}_{13\,1} = G^{\alpha}_{7}$, $w^{\alpha}_{13\,3} = G^{\alpha}_{8}$, $w^{\alpha}_{14\,1} = G^{\alpha}_{9}$, $w^{\alpha}_{14\,4} = G^{\alpha}_{10}$, $w^{\alpha}_{14\,5} = G^{\alpha}_{11}$, $w^{\alpha}_{15\,1} = G^{\alpha}_{12}$, $w^{\alpha}_{15\,5} = G^{\alpha}_{13}$, $w^{\alpha}_{16\,2} = G^{\alpha}_{14}$, $w^{\alpha}_{17\,2} = G^{\alpha}_{15}$, $w^{\alpha}_{18\,1} = G^{\alpha}_{16}$, $w^{\alpha}_{18\,4} = G^{\alpha}_{17}$, $w^{\alpha}_{18\,5} = G^{\alpha}_{18}$,
otherwise $w^{\alpha}_{mi} = 0.0$,
$c_{5\,knee} = G^{c}_{1}$, $c_{6\,knee} = G^{c}_{2}$, $c_{2\,hip} = G^{c}_{3}$, $c_{1\,hip} = G^{c}_{4}$, $c_{1\,knee} = G^{c}_{5}$, $c_{9\,knee} = G^{c}_{6}$, $c_{8\,knee} = G^{c}_{7}$, $c^{'}_{9\,knee} = G^{c^{'}}_{1}$ $c^{'}_{8\,knee} = G^{c^{'}}_{2}$
$c_{14\,knee} = G^{c}_{8}$, $c_{15\,knee} = G^{c}_{9}$, $c_{11\,hip} = G^{c}_{10}$, $c_{10\,hip} = G^{c}_{11}$, $c_{10\,knee} = G^{c}_{12}$, $c_{18\,knee} = G^{c}_{13}$, $c_{17\,knee} = G^{c}_{14}$, $c^{'}_{18\,knee} = G^{c^{'}}_{3}$ $c^{'}_{17\,knee} = G^{c^{'}}_{4}$
otherwise $c_{mi} = c^{'}_{mi} = 0.0$,

$$
\begin{aligned}
\mathrm{POS}_1 &= G^{\mathrm{POS}}_1(-5.0\theta_{\text{trunk}} + 1.0\dot{\theta}_{\text{trunk}}),\\
\mathrm{POS}_2 &= G^{\mathrm{POS}}_2(1.0\theta_{\text{trunk}} - 1.0\dot{\theta}_{\text{trunk}}),\\
\mathrm{POS}_4 &= G^{\mathrm{POS}}_3(-0.5\mathrm{COM}^v),\\
\mathrm{POS}_7 &= G^{\mathrm{POS}}_4(0.1\mathrm{COM}^v),\\
\mathrm{POS}_8 &= G^{\mathrm{POS}}_5(0.01\mathrm{COM}^v),\\
\mathrm{POS}_{10} &= G^{\mathrm{POS}}_6(-5.0\theta_{\text{trunk}} + 1.0\dot{\theta}_{\text{trunk}}),\\
\mathrm{POS}_{11} &= G^{\mathrm{POS}}_7(1.0\theta_{\text{trunk}} - 1.0\dot{\theta}_{\text{trunk}}),\\
\mathrm{POS}_{13} &= G^{\mathrm{POS}}_8(-0.5\mathrm{COM}^v),\\
\mathrm{POS}_{16} &= G^{\mathrm{POS}}_9(0.1\mathrm{COM}^v),\\
\mathrm{POS}_{17} &= G^{\mathrm{POS}}_{10}(0.01\mathrm{COM}^v),\\
\text{otherwise } \mathrm{POS}_m &= 0.0,
\end{aligned}
$$

where θ_{trunk} and $\dot{\theta}_{\text{trunk}}$ are the trunk angle and the trunk angular velocity, respectively. COM^v indicates the center of mass velocity.

(b) Pathological locomotion model

All parameters except the following are identical to the normal locomotion model.
$w^{\text{condition}}_1, \cdots, w^{\text{condition}}_6 = 0.8, w^{\text{condition}}_7, \cdots, w^{\text{condition}}_9 = 0.6$.
